# Supplementary material for: miR551b Regulates Colorectal Cancer Progression by Targeting the ZEB1 Signaling Axis
Source: Cancers (Basel). 2019 May 27;11(5):735. doi: 10.3390/cancers11050735 (PMC6563032; doi:10.3390/cancers11050735)
Supplement: Supplementary file 1 [file cancers-11-00735-s001.pdf]

Table S1: Primers used for realtime qPCR

No.	Target Gene	Sequence (5′–3′)
1	ZEB1-F	GATGTGAATGCGAGTCAGATGC
	ZEB1-R	CTGGTCCTCTTCAGGTGCC
2	B2M-F	TGAAGCTGACAGCATTCGG
	B2M-R	CTGCTGGATGACGTGAGTAAA
3	E-CAD-F	TTGCACCGGTCGACAAAGGAC
	E-CAD-R	TGGATTCCAGAAACGGAGGCC
4	N-CAD-F	GGTGGAGGAGAAGAAGACCAG
	N-CAD-R	GGCATCAGGCTCCACAGT
5	SNAIL1-F	CTGGGTGCCCTCAAGATGCA
	SNAIL1-R	CCGGACATGGCCTTGTAGCA
6	VIMENTIN-F	ACCCGCACCAACGAGAAGGT
	VIMENTIN-R	ATTCTGCTGCTCCAGGAAGCG

Table S2: Clinical pathological characteristics.

NO	Age	Gender	TNM stage	V. Inv.	L. Inv.	PN. Inv.
1	40	F	IIA	0	0	1
2	70	F	IIIC	0	1	1
3	69	M	IIIB	0	1	1
4	72	M	IIA	0	0	1
5	58	M	I	1	1	0
6	70	F	IIIB	0	1	0
7	77	M	IIA	0	0	0
8	82	F	IIB	0	0	1
9	85	M	I	0	0	0
10	59	M	I	0	0	0

V.Inv.: Vescular invasion, L.Inv.: Lymphovascular invasion, PN.Inv.: Perineural invasion.

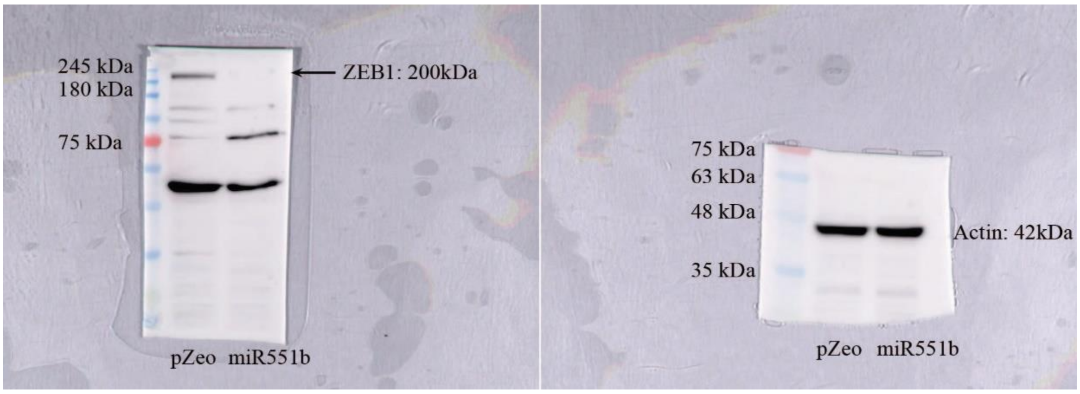

Figure S1: The whole membrane with all molecular weight markers on the western blot. ZEB1 expression was evaluated in control (pZeo)- or miR551b- transduced SW620 cells by immunoblotting. ACTIN was used as the loading control.
